# Evaluating the health system financing of the Eastern Mediterranean Region (EMR) countries using Grey Relation Analysis and Shannon Entropy

**DOI:** 10.1186/s12962-018-0151-6

**Published:** 2018-09-17

**Authors:** Kimia Pourmohammadi, Payam Shojaei, Hamed Rahimi, Peivand Bastani

**Affiliations:** 1 Health Care Management, School of Management and Medical Informatics, Shiraz University of Medical Sciences, Shiraz, Iran; 2 Department of Management, Shiraz University, Shiraz, Iran; 3 Health Services Management Research Center, Institute for Futures Studies in Health, Kerman University of Medical Sciences, Kerman, Iran; 4 Health Human Resources Research Center, School of Management and Medical Informatics, Shiraz University of Medical Sciences, Shiraz, Iran; 5 Kerman University of Medical, haft baghe alavi Blvd, Kerman, Iran

**Keywords:** Health financing, Expenditure, Out of Pocket, Eastern Mediterranean Region, Grey Relation Analysis

## Abstract

**Background:**

Sufficient and sustainable financing of the health system is essential for improving the health of the community. The health systems financing of the EMR countries is facing the challenge. Assessment and ranking of healthcare financing can help identify and resolve some challenges of health systems. So, the aim of this study is to evaluate and rank the condition of the health sector financing in the EMR countries.

**Methods:**

This study was a cross-sectional study. The data was of secondary type, extracted from the official WHO and World Bank data. The six healthcare financing indicators in a 10-year interval (2005–2014) in 19 EMR countries analyzed using Grey Relation Analysis and Shannon Entropy.

**Results:**

On average, the countries in the EMR region spent 4.87% of their GDP on the health sector. Jordan and Qatar allocated the highest (8.313) and the lowest (2.293) percentages of their GDP to the health sector, respectively. The results showed That Qatar was in a better condition than other EMR countries during 2005–2014 in terms of the health system financing and earned the first rank. After that, the UAE and Kuwait were ranked second and third.

**Conclusions:**

There is a lot of inequality among the EMR countries in terms of health financing. However, our findings confirmed that only increasing the total health expenditure in a country would not improve its financing status compared to other countries, but it also depends on financing methods.

**Electronic supplementary material:**

The online version of this article (10.1186/s12962-018-0151-6) contains supplementary material, which is available to authorized users.

## Background

Providing community health is a key element in economic growth of countries. However, increasing the burden of diseases reduces the pace of economic growth. Improving people’s health is not only an objective to improve the quality of life, but also has a positive impact on economic development of the country [1]. On the other hand, economic growth contributes to the improvement of health indicators. For example, a 5% increase in Gross Domestic Product (GDP), on average, may result in a 1% reduction in infant mortality rates [2]. So there is a mutual relationship between health and economy.

Governments need to have powerful health systems to improve the health of their communities, one of the most important aspects of which is its financing [3]. Sufficient and sustainable financing of the health system is essential for improving the health of the community and achieving the Millennium Development Goals [4, 5]. The World Health Organization (WHO) has considered and emphasized financing arrangements of the health system, and in 2010, encouraged countries to ensure adequate expenditures in the health sector and improve the efficiency of expenditures in order to have access to universal health coverage [6, 7]. But the rapid growth of health expenditures is a major concern for families and governments, especially in developing countries [8–10].

Evidence shows that health expenditures are allotted %1.5 to %13 of any country’s GDP [4]. As the most expensive health system in the world, the United States has spent one-sixth of its final goods and service expenditures on the health sector in the second decade of the twenty-first century [11]. Meanwhile, the health systems of the EMR countries are facing the challenge of increased health expenditures [4]. For instance, Iran’s health expenditure indicator has increased 71 times in the last 20 years [12]. The study by Ahmadi et al. [4] in 2013 showed that among the EMR countries, Pakistan and Qatar had the lowest and the highest expenditures in the health sector, respectively. The EMR is one of the six WHO regions which is expanded from Pakistan to Morocco, covering 22 countries with an estimated population of 645 million [13].

Therefore, as health expenditures is a major part of governments’ expenditures and one of the key indicators of governments’ commitment to the health of the communities under their coverage, they are looking for appropriate policies and strategies to control or reduce these expenditures [12, 14]. A comprehensive study of healthcare financing and expenditures can help identify and resolve some challenges of health systems [4]. On the other hand, assessment and ranking of countries’ healthcare financing may provide the policymakers with new ideas and approaches to improve the performance of the health system, particularly economic performance [15]. Ranking has always existed, but the large amount of data in the present era has made ranking inevitable. Governments and organizations tend to know how to invest their resources and be distinguished compared to their competitors. Ranking can help identify distinctions and make decision-making easier [16].

Given that there are many indicators in the field of health system financing, its evaluation is multidimensional and complex. The ranking and multi-criteria decision-making (MCDM) approach plays an important role in solving multidimensional and complex problems [17]. There are many techniques for solving multidimensional and complex problems such as Simple Additive Weighting, Analytic Hierarchy Process, Analytic Network Process, Fuzzy theory, Goal Programming, Data Envelopment Analysis, TOPSIS, VICOR, ELECTRE, PROMETHEE, Operational Competitiveness Rating, and Grey Relational Analysis (GRA) [18–20]. Previous studies have examined and compared these methods in detail. Each of these methods has strengths and weaknesses [18]. It is not easy to determine which method is more reliable and logical, but it is believed that the technique should be chosen to be more objective and more efficient in solving the problem. Compared to other methods, GRA is a simple, meaningful, flexible and easy to computing and understand, which has been used in the financial field as well [20, 21].

GRA is one of the most well-known methods for ranking, decision-making and evaluating performance, and is widely used in solving multivariate problems [22, 23]. In the event of poor, limited, and unreliable information, GRA can be useful and effective for evaluating and ranking [24]. GRA is based on Grey System Theory, which was first introduced by Deng in 1982 and measures the relations within a series of discrete data [22, 25, 26]. Grey Relation refers to measuring the changes of the relations between two variables that occur in a system over time. The GRA method is used to measure the relations between variables when their development process is either homogeneous or heterogeneous [25, 27]. GRA is a useful method for solving problems under the conditions of uncertainty and multiple characteristics, and does not require large sample sizes and classical normal distribution [24, 25, 28].

Hence, the GRA approach was used in the present study to evaluate and rank the condition of the health sector financing in the EMR countries.

## Methods

The data in this study are of secondary type, extracted from the official WHO and World Bank data. This study examined and analyzed the six healthcare financing indicators in a 10-year interval (2005–2014) in 19 EMR countries. The 6 indicators include Total Health Expenditure (THE) as % of Gross Domestic Product (GDP) (C1), General Government Health Expenditure (GGHE) as % of Total Government Expenditure (TGE) (C2), Out of Pocket (OOP) as % of THE (C3), THE per capita (current US$) (C4), Public Health Expenditure (PHE) as % of THE (C5), and Out of Pocket expenditure (OOP) as % of Private Health Expenditure (PvtHE) (C6), selected by experts. They are the most widely used indicators of countries’ health economy, so that the WHO and the World Bank use them for annual assessment of countries’ health economy status.

According to WHO classification, EMR countries include: Afghanistan, Bahrain, Egypt, Iran, Iraq, Jordan, Kuwait, Lebanon, Libya, Morocco, Oman, Pakistan, Palestine, Qatar, Saudi Arabia, Sudan, South Sudan, Somalia, Syrian, Tunisia, United Arab Emirates (UAE), and Yemen. Three countries (Palestine, South Sudan, and Somalia) were excluded from the study due to the lack of complete and reliable data.

The GRA method was used to evaluate and rank the remaining countries. It included the following 7 stages [23, 28]:

1. Recognition of the alternatives and indicators: In this study, the items were the EMR countries except Palestine, Somalia and South Sudan (19 countries), and the six healthcare financing indicators mentioned above formed the performance evaluation indicators.
2. Making of the performance matrix: At this stage, a performance matrix was created, in which the rows and the columns were respectively the alternatives (M) and the indicators (N).
  $$Z_{M \times N} = \left[ {\begin{array}{*{20}c} {Z_{11} } & \cdots & {Z_{1j} } \\ \vdots & \ddots & \vdots \\ {Z_{i1} } & \cdots & {Z_{MN} } \\ \end{array} } \right]$$
   The Z_ij_ element represented the actual value of the ith alternative in the jth indicator.
3. Generation of the normalized matrix: Since the indicators did not have the same nature and scale, the performance matrix was normalized. Thus, the values of the performance matrix were converted to the numbers ranged from 0 to 1. For this purpose, the following two formulas were used: the first was for positive indicators (larger is better), and the second was for negative ones (smaller is better):
  1 $$Z'_{ij} = \frac{{Z_{ij} - \hbox{min} \left\{ {Z_{ij} } \right\}}}{{\hbox{max} \left\{ {Z_{ij} } \right\} - \hbox{min} \left\{ {Z_{ij} } \right\}}}$$
  2 $$Z'_{ij} = \frac{{\hbox{min} \left\{ {Z_{ij} } \right\} - Z_{ij} }}{{\hbox{max} \left\{ {Z_{ij} } \right\} - \hbox{min} \left\{ {Z_{ij} } \right\}}}$$
  In this study, C3 and C6 indicators were negative and the rest were positive.
4. Construction of the reference sequence: Reference Sequence (R_j_) refers to the ideal solution to solve the problem with the best performance for each indicator. The reference sequence was obtained in the normalized matrix by taking into account the best normalized value of each indicator.
  3 $$R_{j} = \max {{_{i = 1}}} \left\{ {Z^{\prime}{{_{ij}}} } \right\}$$
5. Construction of the difference matrix: A difference matrix is created by the difference between the entries of the normalized matrix and the reference sequence. Each indicator’s entries were subtracted from the reference of the same indicator.
  4 $$\Delta_{ij} = \left| {R_{j} - Z'_{ij} } \right|$$
  $$\Delta_{M \times N} = \left[ {\begin{array}{*{20}c} {\Delta_{11} } & {\Delta_{12} } & \cdots & {\Delta_{1j} } \\ {\Delta_{21} } & {\Delta_{22} } & \cdots & {\Delta_{2j} } \\ \vdots & \vdots & \cdots & \vdots \\ {\Delta_{i1} } & {\Delta_{i2} } & \cdots & {\Delta_{MN} } \\ \end{array} } \right]$$
6. Definition of the grey relational coefficient: Next, the following formula was used to calculate the grey relational coefficient:
  5 $$\gamma_{ij} = \frac{{min_{i} {\mkern 1mu} min_{j} {\mkern 1mu} \Delta_{ij} + {\mkern 1mu} \rho \,max_{i} {\mkern 1mu} max_{j} \Delta_{ij} }}{{\Delta_{ij} + {\mkern 1mu} \rho \,{\mkern 1mu} max_{i} {\mkern 1mu} max_{j} {\mkern 1mu} \Delta_{ij} }}$$
  In this formula, ρ is the Coefficient of Determination whose value is ranged from 0 to 1 and is usually considered 0.5, because it provides moderate differentiation effects and good stability. The smaller the ρ value, the higher its determination capability will be. In this study, its value was considered 0.5.
7. Computing of the grey relational grade: Finally, the grey relational grade was calculated using the following formula:
  6 $$\gamma_{i} = \mathop \sum \limits_{j = 1}^{n} \left( {w_{j} \times \gamma_{ij} } \right), \mathop \sum \limits_{j = 1}^{n} w_{j} = 1$$
  The grey relational grade is the total weight of the grey relational coefficients, which indicates the correlation between the reference sequence and the sequence of the ith alternative. The Shannon Entropy technique was used to calculate the weight of the indicators. In this technique, the P_ij_ matrix was calculated as follows based on the initial data [28]:
  7 $$P_{ij} = \frac{{z_{ij} }}{{\mathop \sum \nolimits_{i = 1}^{m} r_{ij} }}$$
  Then from the P_ij_ series, a value with the symbol of E_j_ was calculated per indicator.
  8 $$E_{j} = - K \mathop \sum \limits_{i = 1}^{m} \left[ {P_{ij} \times Ln P_{ij} } \right]$$
  As K was a constant positive value, for providing 1 ≥ E ≥ 0. ${\text{K}} = \frac{1}{Lnm}$, given that m = 19 in this study, the K value was calculated to be 0.34. From the data generated for the jth indicator, the degree of deviation (d_j_) was calculated as follows:
  9 $$d_{j} = 1 - E_{j}$$
  Finally, the weights of the indicators (W_j_) were calculated using the following formula:
  10 $$W_{j} = \frac{{d_{j} }}{{\mathop \sum \nolimits_{j = 1}^{n} d_{j} }}$$
  All the calculations above were done separately for each year and for the mean data of 2005–2014 using the Microsoft Office Excel software 2013.

## Results

This study evaluated the performance of the EMR countries’ health system financing, using GRA and Shannon Entropy. As stated above, the analyses and rankings were conducted both on an annual basis and for the average period of 2005–2014. However, due to the large number of tables and the high volume of information, the tables showing the average period of 10 years are presented here. The final results of the annual analyses, including the grey relational grade and the ranks of the countries are presented in Tables 3 and 4.

Table 1 shows the mean healthcare financing indicators of the EMR countries during the years 2005–2014, which is also the GRA performance matrix. On average, the countries in the region spent 4.87% of their GDP on the health sector. Furthermore, in these countries, Out of Pocket expenditure (OOP) accounted for 40% of total health expenditure (THE) and 84% of private expenditure on health (PvtHE).

**Table 1:** Mean healthcare financing indicators of the EMR countries during the years 2005–2014

Countries	Indicator					
	C1	C2	C3	C4	C5	C6
Afghanistan	8.188	9.32	76.05	42.177	23.626	99.574
Bahrain	3.734	9.55	20.50	818.453	68.617	65.154
Egypt	5.132	5.84	57.02	120.193	39.789	94.793
I.R Iran	6.728	13.20	52.24	360.840	38.975	85.592
Iraq	4.326	4.94	31.59	174.001	68.412	100
Jordan	8.313	15.52	27.73	311.012	64.768	77.424
Kuwait	2.612	5.81	15.27	1147.765	83.137	90.543
Lebanon	7.598	10.18	41.90	569.380	42.449	72.733
Libya	3.447	5.22	32.00	363.324	67.995	100
Morocco	5.669	5.95	57.70	156.102	33.943	87.388
Oman	2.624	5.75	10.19	488.506	82.828	59.086
Pakistan	2.997	4.48	62.07	31.350	29.468	87.952
Qatar	2.293	6.22	13.16	1733.858	82.737	76.172
Saudi Arabia	3.726	7.60	16.98	737.907	70.650	57.889
Sudan	6.908	9.88	66.91	99.842	29.227	94.458
Syrian	3.483	5.88	52.83	77.707	47.166	100
Tunisia	6.381	13.60	38.36	256.790	55.650	86.525
UAE	3.240	8.72	21.65	1312.441	68.522	67.933
Yemen	5.211	4.29	70.81	65.309	27.722	97.923
Mean	4.874	7.996	40.260	466.682	53.983	84.271

Indicators: C1: Total Health Expenditure (THE) as % of Gross Domestic Product (GDP), C2: General Government Health Expenditure (GGHE) as % of Total Government Expenditure (TGE), C3: Out of Pocket (OOP) as % of THE, C4: THE per capita (current US$), C5: Public Health Expenditure (PHE) as % of THE, C6: Out of Pocket expenditure (OOP) as % of Private Health Expenditure (PvtHE)

On average, Jordan and Qatar allocated the highest (8.313) and the lowest (2.293) percentages of their GDP to the health sector, respectively. However, in terms of the THE per capita, Qatar and Pakistan had the highest (1733.858 US$) and the lowest (31.35 US$) THE per capita, respectively. Also, the highest and the lowest OOPs as the percentages of THE were respectively those of Afghanistan (76.046) and Oman (10.18). While Kuwait provided an average of 83.137% of its own THE through the public, Afghanistan had the lowest rate of 23.626%. In Iran, on average, 38.97% of THE was supplied through the public during the years of this study (Table 1).

The data analysis and the evaluation of the financing performance of the EMR countries based on the data in Table 1, and also the GRA methodology are provided in the following. First of all, the performance matrix was normalized using Eq. 1 and Eq. 2 formulas. The aim of normalizing the performance matrix was to convert the original data into a comparable sequence. After normalization, the reference sequence was defined by Eq. 3 formula (Additional file 1: Table S1). Then, the difference matrix was created by calculating the difference between the entries of the normalized matrix and its reference value based on Eq. 4 formula (Additional file 1: Table S2). In the next step, the grey relational coefficients were obtained through Eq. 5 formula (Additional file 1: Table S3).

In order to calculate the grey relational grade, the weights of the indicators were first calculated using Eq. 7 to Eq. 10 formulas of Shannon Entropy. According to the Shannon Entropy calculations, the most important indicator in the evaluation of the health system financing of the EMR countries was THE per capita. In contrast, the indicator of OOP percentage of private health expenditures had the lowest weight compared to other indicators (Table 2).

**Table 2:** Shannon Entropy calculations for healthcare financing indicators of the EMR countries

Entropy calculations	Indicators					
	C1	C2	C3	C4	C5	C6
E_j_	0.9747	0.9752	0.9536	0.8468	0.9769	0.9963
d_j_	0.02526	0.02477	0.04636	0.15312	0.02307	0.00368
W_j_	0.09144	0.08965	0.16781	0.55425	0.08352	0.01333

Finally, the grey grade of health system financing of the EMR countries was calculated using Eq. 6 formula. Table 3 shows the grey relational grade of the countries both for the years 2005–2014 and for each single year of conducting this study. The grey relational grade indicates the degree of correlation between the status of an alternative and the reference state (Ideal state). The higher the obtained value, a more favorable status the item will have. According to Table 3, Qatar had the highest average grey grade (0.8619) and was in a more favorable status than other EMR countries in terms of financing the health system. In contrast, the average 10-year period showed that Pakistan had the lowest grey grade (0.3485) and the poorest health system financing status. However, an annual investigation showed that Pakistan had the most unfavorable status from 2005 to 2011, and Yemen had the worst healthcare financing situation from 2012 to 2014 (Table 3).

**Table 3:** Grey relational grade of the EMR countries in healthcare financing during the years 2005–2014

Countries	Year										
	2005	2006	2007	2008	2009	2010	2011	2012	2013	2014	Mean 10 years
Afghanistan	0.3824	0.3769	0.3606	0.4167	0.4155	0.4498	0.4023	0.4305	0.4074	0.4098	0.4042
Bahrain	0.5069	0.5127	0.5440	0.5546	0.5508	0.5498	0.5393	0.5241	0.5467	0.5629	0.5406
Egypt	0.3800	0.3853	0.3903	0.3871	0.3711	0.3724	0.3787	0.3777	0.3791	0.3805	0.3769
I.R Iran	0.4143	0.4228	0.4436	0.4379	0.4359	0.4605	0.4592	0.5036	0.4859	0.4770	0.4485
Iraq	0.4144	0.3994	0.4379	0.4520	0.4448	0.4410	0.4467	0.4247	0.4325	0.4185	0.4281
Jordan	0.5075	0.4904	0.5318	0.5851	0.5959	0.6067	0.6056	0.5808	0.5127	0.5163	0.5566
Kuwait	0.5657	0.5924	0.5949	0.5915	0.7656	0.6295	0.6635	0.6149	0.5961	0.6222	0.6268
Lebanon	0.5096	0.5068	0.5275	0.5087	0.4692	0.4730	0.4690	0.4769	0.4653	0.4595	0.4825
Libya	0.4220	0.4216	0.4419	0.4328	0.4366	0.4458	0.4302	0.4731	0.4510	0.4538	0.4397
Morocco	0.3760	0.3821	0.3969	0.3951	0.3793	0.3857	0.3878	0.3870	0.3803	0.3798	0.3818
Oman	0.5160	0.5113	0.5393	0.5285	0.5489	0.5641	0.5549	0.5388	0.5640	0.5860	0.5525
Pakistan	0.3529	0.3516	0.3588	0.3542	0.3428	0.3503	0.3496	0.3579	0.3585	0.3572	0.3485
Qatar	0.8870	0.8801	0.8644	0.8279	0.8252	0.7887	0.8244	0.8607	0.8764	0.8767	0.8619
Saudi Arabia	0.5075	0.5260	0.5349	0.5028	0.5196	0.5160	0.5566	0.5544	0.5669	0.5820	0.5401
Sudan	0.3661	0.3682	0.3859	0.4437	0.3921	0.4172	0.4250	0.4161	0.4143	0.4049	0.3931
Syrian	0.3922	0.3839	0.3953	0.3843	0.3686	0.3682	0.3673	0.3658	0.3663	0.3665	0.3716
Tunisia	0.4492	0.4537	0.4661	0.4574	0.4501	0.4813	0.4863	0.4782	0.4748	0.4619	0.4647
UAE	0.5445	0.5600	0.5985	0.6719	0.6932	0.7173	0.7053	0.6228	0.6380	0.6445	0.6348
Yemen	0.3669	0.3676	0.3702	0.3709	0.3470	0.3510	0.3520	0.3573	0.3537	0.3493	0.3550

In the end, based on the countries’ gray relational grade in Table 3, the EMR countries were ranked on the basis of the health system financing during 2005–2014. Table 4 shows that Qatar was in a better condition than other EMR countries during 2005–2014 in terms of the health system financing and earned the first rank. After that, the UAE and Kuwait were ranked second and third. In contrast, Pakistan and Yemen were ranked the last ones, respectively. Iran and Sudan, with a promotion of 4 ranks in 2014 compared to 2005, had the highest promotion. Iran was ranked 12 in 2005 but was promoted to 8th in 2014, with an average rank of 10. In contrast, Lebanon and Syria, with a relegation of 5 and 3 ranks, respectively, had more unfavorable conditions in 2014 compared to 2005 (Table 4).

**Table 4:** Ranking of the EMR countries in healthcare financing during the years 2005–2014

Countries	Year										
	2005	2006	2007	2008	2009	2010	2011	2012	2013	2014	Mean 10 years
Qatar	1	1	1	1	1	1	1	1	1	1	1
UAE	3	3	2	2	3	2	2	2	2	2	2
Kuwait	2	2	3	3	2	3	3	3	3	3	3
Jordan	7	8	7	4	4	4	4	4	7	7	4
Oman	4	6	5	6	6	5	6	6	5	4	5
Bahrain	8	5	4	5	5	6	7	7	6	6	6
Saudi Arabia	6	4	6	8	7	7	5	5	4	5	7
Lebanon	5	7	8	7	8	9	9	10	10	10	8
Tunisia	9	9	9	9	9	8	8	9	9	9	9
I.R Iran	12	10	10	12	12	10	10	8	8	8	10
Libya	10	11	11	13	11	12	12	11	11	11	11
Iraq	11	12	12	10	10	13	11	13	12	12	12
Afghanistan	14	16	18	14	13	11	14	12	14	13	13
Sudan	18	17	16	11	14	14	13	14	13	14	14
Morocco	16	15	13	15	15	15	15	15	15	16	15
Egypt	15	13	15	16	16	16	16	16	16	15	16
Syrian	13	14	14	17	17	17	17	17	17	17	17
Yemen	17	18	17	18	18	18	18	19	19	19	18
Pakistan	19	19	19	19	19	19	19	18	18	18	19

## Discussion

According to the findings of this study, indefinite values were obtained through GRA for the health system financing of the EMR countries. The GRA method showed that there were great differences between the health systems financing of the EMR countries. The findings indicated that, based on the indicators under study, Qatar was nearly in a favorable condition. In contrast, Pakistan’s health system financing needed to be paid more attention and strengthened, because according to the results of the study, it was far away from the favorable condition compared to other EMR countries. However, the negative impact of war and insecurity on the performance of the health system, especially its financing performance, in some countries of the region (Afghanistan, Iraq and Syrian) should not be ignored.

According to the World Bank statistics, Qatar had a gross national income of $ 161 billion and 6.45% GDP growth in 2015, with a population of only 2,569,804 people in 2015. In contrast, Pakistan, with the population of 97,286,333 in 2015 had $ 287 billion gross national income and 5.16% GDP growth [29, 30]. As Table 1 shows, although Pakistan allocated a higher percentage of its GDP to the health sector compared to Qatar, the 38-fold difference in the population of these two countries has led the THE per capita to be 55.3 times more in Qatar than in Pakistan. The study by Ahmadi et al. [4] also showed that during 1995–2011, Qatar and Pakistan were ranked first and last, respectively, in terms of the THE per capita. In his study, Shetty suggested that the low share of health expenditures from GDP in countries like Qatar could be attributed to their low population [31].

The results showed that, on average, about 54% of the THE in the EMR countries was provided through the public sector. Thus, it can be said that the remaining 46% was provided through the private sector. According to the WHO definition, PvtHE refers to the total expenditure on health by private entities including families, commercial insurance, health insurance, nonprofit institutions, and the companies providing or financing health services [32]. But the results showed that 84% of PvtHE was directly paid by households. Therefore, it seems that in these countries, private insurance and non-profit institutions supporting the health sector have not been developed well and have played a minor role in healthcare financing. The results also showed that countries that provided a higher percentage of their PvtHE through direct payments by households (Syria, Libya, Iraq, Afghanistan and Yemen) were ranked lower in the ranking of healthcare financing performance. Shetty stated that one of the causes of high PvtHE in poorer countries was the lack of access to quality public health services [31].

Although private budgets play an important role in health systems, evidence shows that public financing helps countries achieve Universal Health Coverage (UHC) [33]. For this reason, increased government financing in the health sector is highly emphasized [34]. The results showed that in terms of GGHE share of TGE, Jordan, Tunisia and Iran had the highest rates, respectively, and were ranked 4, 10 and 9. On the other hand, Yemen, Pakistan and Iraq had the lowest rates, ranked 18, 19 and 12.

Countries whose average OOP share of THE was over 50%, had poorer healthcare financing performance and were ranked 10 to 19, while the OOP share of THE in the first 5 countries was less than 28%. The Entropy analysis showed that among the indicators under study, the OOP was the second indicator influencing the performance of the health system financing. According to the calculations done in the present study, the average OOP in the EMR countries was 40% for health services during 2005–2014. This amount was 32.1% for the whole world and 17.9% for Organization for Economic Co-operation and Development (OECD) high income countries in 2013 [35].

In countries with high OOP rates, the risk of catastrophic expenditures is high, too. The high OOP for health services imposes a heavy financial burden on households and, in the long term, may result in negative social and economic outcomes, so that it may keep the poor in poverty and push them below the poverty line [36, 37]. Catastrophic and impoverishing health expenditures indicate insufficient financial protection [35]. Sambo et al. [37] suggested that countries should develop healthcare financing models to optimize the use of health resources. To reduce potential catastrophic and impoverishing OOPs, they recommended an increased coverage of prepaid financing mechanisms. Ahmadi et al. [4] too, proposed the allocation of international financial contributions, donations and loans for providing cost-effective health programs in some countries.

Given the fact that health is one of the key factors in the welfare of countries and global economic growth, especially in low-income and lower middle income countries, [38, 39] the EMR countries need to pay more attention to investing and financing their health sectors. In this regard, the WHO considers adequate healthcare financing to be essential for countries to access UHC [7, 40]. UHC is a part of the global commitment to sustainable development goals, one of the main components of which is financial coverage with the aim of ensuring the people’s lack of exposer to difficult decision-making for choosing health services or other essential needs [35, 38]. However, the countries around the world are facing ever-increasing problems with healthcare financing and moving towards UHC [41].

According to the WHO, access to resources, excessive dependency on direct OOP, and inefficient and unfair use of resources are the three most important problems for access to UHC [42]. Although every country has its own challenges, it is worthwhile to use the experiences of other countries to solve common problems [40]. Therefore, it is recommended that countries with an unfavorable condition in the ranking of this study should take advantage of the experiences of other countries to improve their healthcare financing system. Sakha et al. conducted a systematic review in 2017 with the aim of identifying financing policies and strategies for achieving UHC. They categorized the important dimensions of healthcare financing for achieving UHC in 9 groups: stewardship, increasing income and participation methods, risk pooling and financial protection, resource allocation and purchasing, human resources, policy stockholders, policy content, policy context, and policy process [40]. Mehrolhassani et al. concluded that Iran had no significant legal and policy gap in financing for access to UHC. But the major constraints in this country were the ways to implement it and the commitment to laws that had made fundamental challenges to financial protection. To overcome these challenges, they proposed adequate political support and a common understanding among the stakeholders at different levels of policy-making and implementation [43].

## Limitations and suggestions

The limitation of this study was the lack of investigating the outcome indicators of the countries’ health systems. Investigating the outcomes of the health system along with the financing indicators will show the great impact of adequate and proper financing on health outcomes. Therefore, it is suggested that in future studies, the EMR countries be evaluated and ranked based on the outcome indicators of the health system and through the use of GRA. It is also suggested that the method used in this study be applied to rank the countries in other WHO regions, or the income groups (high-income, middle-income and low-income).

## Conclusion

There is a lot of inequality among the EMR countries in terms of health financing. However, our findings confirmed that only increasing the total health expenditure in a country would not improve its financing status compared to other countries, but it also depends on financing methods. Although some countries spend a higher percentage of their GDP on the health system, they have a poorer financing performance due to the high rates of OOP. Thus, the countries with a poor financing status can improve their situation and move towards UHC without increasing their total health expenditure, but by correcting their financing methods.

## Additional file

**Additional file 1: Table S1.** Normalized performance matrix and reference sequence. **Table S2.** The difference matrix. **Table S3.** The grey relational coefficients.

## Abbreviations

WHO: World Health Organization
OECD: Organization for Economic Co-operation and Development
EMR: Eastern Mediterranean Region
MCDM: multi-criteria decision-making
GRA: Grey Relation Analysis
GDP: Gross Domestic Product
UHC: Universal Health Coverage
OOP: Out of Pocket
GGHE: General Government Health Expenditure
TGE: Total Government Expenditure
THE: Total Health Expenditure
PvtHE: Private Health Expenditure
PHE: Public Health Expenditure
UAE: United Arab Emirates
