# Supplementary material for: Evaluating the health system financing of the Eastern Mediterranean Region (EMR) countries using Grey Relation Analysis and Shannon Entropy
Source: Cost Eff Resour Alloc. 2018 Sep 17;16:31. doi: 10.1186/s12962-018-0151-6 (PMC6142403; doi:10.1186/s12962-018-0151-6)
Supplement: Supplementary file 1 — Additional file 1: Table S1. Normalized performance matrix and reference sequence. Table S2. The difference matrix. Table S3. The grey relational coefficients. [file 12962_2018_151_MOESM1_ESM.docx]

Table S1. Normalized performance matrix and reference sequence

| **Indicator**  **Countries** | **C1** | **C2** | **C3** | **C4** | **C5** | **C6** |
| --- | --- | --- | --- | --- | --- | --- |
| Afghanistan | 0.97931928 | 0.448226698 | 0 | 0.00635997 | 0 | 0.010128043 |
| Bahrain | 0.239431239 | 0.468098378 | 0.843382275 | 0.462319797 | 0.756020756 | 0.827474187 |
| Egypt | 0.471603462 | 0.137408662 | 0.288881128 | 0.052183775 | 0.271591039 | 0.123654739 |
| I.R Iran | 0.736798392 | 0.793263233 | 0.361412672 | 0.193532413 | 0.257917891 | 0.342140259 |
| Iraq | 0.337801698 | 0.057476386 | 0.675042893 | 0.083788911 | 0.75256088 | 0 |
| Jordan | 1 | 1 | 0.733681541 | 0.164265122 | 0.691331657 | 0.536109502 |
| Kuwait | 0.052972542 | 0.135002673 | 0.922791941 | 0.655747247 | 1 | 0.224574218 |
| Lebanon | 0.881314264 | 0.524950989 | 0.518409985 | 0.316022226 | 0.316292169 | 0.647517976 |
| Libya | 0.191741001 | 0.082783818 | 0.668711377 | 0.19499126 | 0.74556383 | 0 |
| Morocco | 0.560771416 | 0.147567279 | 0.278556354 | 0.073275716 | 0.173366851 | 0.299486118 |
| Oman | 0.055048919 | 0.129656033 | 1 | 0.268519192 | 0.994809346 | 0.971570239 |
| Pakistan | 0.11695819 | 0.016218143 | 0.212265225 | 0 | 0.098168736 | 0.286102378 |
| Qatar | 0 | 0.171359829 | 0.954889844 | 1 | 0.993288613 | 0.565835843 |
| Saudi Arabia | 0.238135579 | 0.294510782 | 0.896767434 | 0.41500989 | 0.790180942 | 1 |
| Sudan | 0.766615173 | 0.49741579 | 0.138701204 | 0.040230352 | 0.094122411 | 0.13159807 |
| Syrian | 0.197687746 | 0.141507753 | 0.352439228 | 0.027228769 | 0.395563494 | 0 |
| Tunisia | 0.679158154 | 0.829531278 | 0.572250649 | 0.132416639 | 0.538115771 | 0.319989171 |
| UAE | 0.157339579 | 0.394760292 | 0.825921258 | 0.752473189 | 0.754421046 | 0.761498238 |
| Yemen | 0.484692945 | 0 | 0.079576684 | 0.019946513 | 0.068822802 | 0.049322264 |
| Rj | 1 | 1 | 1 | 1 | 1 | 1 |

Table S2. The difference matrix

| **Indicator**  **Countries** | **C1** | **C2** | **C3** | **C4** | **C5** | **C6** |
| --- | --- | --- | --- | --- | --- | --- |
| Afghanistan | 0.02068072 | 0.551773302 | 1 | 0.99364003 | 1 | 0.989871957 |
| Bahrain | 0.760568761 | 0.531901622 | 0.156617725 | 0.537680203 | 0.243979244 | 0.172525813 |
| Egypt | 0.528396538 | 0.862591338 | 0.711118872 | 0.947816225 | 0.728408961 | 0.876345261 |
| I.R Iran | 0.263201608 | 0.206736767 | 0.638587328 | 0.806467587 | 0.742082109 | 0.657859741 |
| Iraq | 0.662198302 | 0.942523614 | 0.324957107 | 0.916211089 | 0.24743912 | 1 |
| Jordan | 0 | 0 | 0.266318459 | 0.835734878 | 0.308668343 | 0.463890498 |
| Kuwait | 0.947027458 | 0.864997327 | 0.077208059 | 0.344252753 | 0 | 0.775425782 |
| Lebanon | 0.118685736 | 0.475049011 | 0.481590015 | 0.683977774 | 0.683707831 | 0.352482024 |
| Libya | 0.808258999 | 0.917216182 | 0.331288623 | 0.80500874 | 0.25443617 | 1 |
| Morocco | 0.439228584 | 0.852432721 | 0.721443646 | 0.926724284 | 0.826633149 | 0.700513882 |
| Oman | 0.944951081 | 0.870343967 | 0 | 0.731480808 | 0.005190654 | 0.028429761 |
| Pakistan | 0.88304181 | 0.983781857 | 0.787734775 | 1 | 0.901831264 | 0.713897622 |
| Qatar | 1 | 0.828640171 | 0.045110156 | 0 | 0.006711387 | 0.434164157 |
| Saudi Arabia | 0.761864421 | 0.705489218 | 0.103232566 | 0.58499011 | 0.209819058 | 0 |
| Sudan | 0.233384827 | 0.50258421 | 0.861298796 | 0.959769648 | 0.905877589 | 0.86840193 |
| Syrian | 0.802312254 | 0.858492247 | 0.647560772 | 0.972771231 | 0.604436506 | 1 |
| Tunisia | 0.320841846 | 0.170468722 | 0.427749351 | 0.867583361 | 0.461884229 | 0.680010829 |
| UAE | 0.842660421 | 0.605239708 | 0.174078742 | 0.247526811 | 0.245578954 | 0.238501762 |
| Yemen | 0.515307055 | 1 | 0.920423316 | 0.980053487 | 0.931177198 | 0.950677736 |

Table S3. The grey relational coefficients

| **Indicator**  **Countries** | **C1** | **C2** | **C3** | **C4** | **C5** | **C6** |
| --- | --- | --- | --- | --- | --- | --- |
| Afghanistan | 0.96028138 | 0.475387613 | 0.333333333 | 0.334752678 | 0.333333333 | 0.33559931 |
| Bahrain | 0.396646352 | 0.484542314 | 0.761478073 | 0.48184402 | 0.67206176 | 0.743465887 |
| Egypt | 0.48619378 | 0.366947878 | 0.412841391 | 0.345347698 | 0.40703057 | 0.36328094 |
| I.R Iran | 0.655134888 | 0.707476989 | 0.439140668 | 0.38271137 | 0.402549877 | 0.431831233 |
| Iraq | 0.430219179 | 0.346614776 | 0.606092118 | 0.353054713 | 0.668950803 | 0.333333333 |
| Jordan | 1 | 1 | 0.652470255 | 0.37432578 | 0.61830045 | 0.518731122 |
| Kuwait | 0.345535945 | 0.366301084 | 0.866238771 | 0.592239704 | 1 | 0.392025947 |
| Lebanon | 0.808164745 | 0.512794736 | 0.509377634 | 0.422305225 | 0.422401531 | 0.586522632 |
| Libya | 0.382187319 | 0.352804326 | 0.601475813 | 0.383139197 | 0.662746592 | 0.333333333 |
| Morocco | 0.532351771 | 0.369704158 | 0.409351673 | 0.350453136 | 0.376893944 | 0.416488312 |
| Oman | 0.346032476 | 0.364871895 | 1 | 0.40601526 | 0.989725355 | 0.946199545 |
| Pakistan | 0.36152197 | 0.336976758 | 0.388278712 | 0.333333333 | 0.356676308 | 0.41189635 |
| Qatar | 0.333333333 | 0.376324614 | 0.917245798 | 1 | 0.986755011 | 0.535237834 |
| Saudi Arabia | 0.396239082 | 0.414769367 | 0.828867718 | 0.460833694 | 0.704404868 | 1 |
| Sudan | 0.681770308 | 0.498711226 | 0.367296292 | 0.342519795 | 0.355649741 | 0.365389721 |
| Syrian | 0.3839325 | 0.3680551 | 0.435706772 | 0.33949604 | 0.452719552 | 0.333333333 |
| Tunisia | 0.609130738 | 0.745746943 | 0.538938669 | 0.365608426 | 0.519813076 | 0.423724925 |
| UAE | 0.37239498 | 0.452390551 | 0.741753106 | 0.668872331 | 0.670619788 | 0.677046455 |
| Yemen | 0.492461859 | 0.333333333 | 0.352007739 | 0.337825629 | 0.349362749 | 0.344666488 |
